# PATHOPHYSIOLOGY OF POSTTRAUMATIC ANKLE OSTEOARTHRITIS: A MULTICENTER PERSPECTIVE

**DOI:** 10.1590/1413-785220243203e282286

**Published:** 2024-07-22

**Authors:** Alexandre Leme Godoy-Santos, Cesar de Cesar, Simone Santini, Mario Herrera-Pérez, Victor Valderrabano, Stefan Rammelt

**Affiliations:** 1. Universidade de São Paulo, Faculdade de Medicina, Hospital das Clínicas HC-FMUSP, Laboratório Prof Manlio Mario Marco Napoli, São Paulo, SP, Brazil.; 2. Hospital Israelita Albert Einstein, Sao Paulo, SP, Brazil.; 3. Duke University, Department of Orthopedic Surgery, Durham, NC, USA.; 4. Department of Orthopaedic and Trauma Surgery, University Campus Bio-Medico of Rome, Rome, Italy.; 5. Hospital Universitario de Canarias, Orthopaedic Department, Foot and Ankle Unit, Tenerife, Spain.; 6. Swiss Ortho Center, Swiss Medical Network, Schmerzklinik Basel, Basel, Switzerland.; 7. UniversitätsCentrum für Orthopädie und Unfallchirurgie, Universitätsklinikum Carl Gustav Carus, Dresden, Germany.

**Keywords:** Ankle Osteoarthritis, Pathophysiology, Molecular Biology, Osteoartrite do Tornozelo, Fisiopatologia, Biologia Molecular

## Abstract

Besides the acute injury and trauma-induced macroscopic alterations, the evolution to posttraumatic ankle osteoarthritis (PTOA) is a complex process progressing at the tissue and molecular level. Furthermore, changes in the molecular pathways affect chondrocyte viability. Treatment modalities for PTOA focal or confined disease include innovative techniques. Objective: Our purpose is to increase medical awareness based on scientific evidence of pathophysiology, molecular biology, and treatment of post-traumatic ankle osteoarthritis. Methods: To support the perspectives of the experts, evidence from the scientific literature respected the PRISMA guidelines and the PICOS search strategy was used. We included case-control, cohort, experimental studies and case reports, written in English. Results: The authors were homogeneously exposed to 282 selected abstracts and 114 full articles directly related to post-traumatic osteoarthritis after malleolar fractures. Conclusion: The pathophysiological factors involved in posttraumatic ankle osteoarthritis, such as biological, structural, mechanical, and molecular changes must be studied together, as the interaction between these factors determines the risk of progression of PTOA. Inhibition of a single catabolic molecule or cascade probably is not sufficient to alter the natural progression of the pathological process. *Evidence level V, expert opinion.*

## RESUMO

*A evolução para a osteoartrite pós-traumática do tornozelo (PTOA) a partir da lesão aguda e das alterações macroscópicas induzidas pelo trauma é um processo complexo, que progride em nível tecidual e molecular. Além disso, as alterações nas vias moleculares afetam a viabilidade dos condrócitos. As modalidades focais ou confinadas de tratamento para PTOA incluem técnicas inovadoras. Objetivo: Nosso objetivo é aumentar a conscientização médica, com base em evidências científicas de fisiopatologia, biologia molecular e tratamento da osteoartrite pós-traumática do tornozelo. Métodos: Para o embasamento das perspectivas dos autores experts, as evidências da literatura científica respeitaram as diretrizes Prisma e a estratégia de busca Picos foi empregada. Incluímos estudos de caso-controle, de coorte, experimentais e relatos de caso, escritos em inglês. Resultados: Os autores foram expostos de forma homogênea a 282 resumos e 114 artigos completos, diretamente relacionados à osteoartrite pós-traumática após fraturas maleolares. Conclusão: Os fatores fisiopatológicos envolvidos na osteoartrite pós-traumática do tornozelo, como alterações biológicas, estruturais, mecânicas e moleculares, devem ser estudados em conjunto, pois a interação entre esses fatores determina o risco de progressão da PTOA. A inibição de uma única molécula catabólica ou cascata provavelmente não é suficiente para alterar a progressão natural do processo patológico.* *Nível de evidência V, opinião do especialista.*

## INTRODUCTION

 Besides the acute injury and trauma-induced macroscopic alterations, the evolution to posttraumatic ankle osteoarthritis (PTOA) is a complex process progressing at the tissue and molecular level. ^1^ ^-^ ^6^ Furthermore, changes to apoptosis pathways, like caspase inhibition, or necrosis progression via antagonist of reactive oxidant species (anti-ROS) are thought to affect chondrocyte viability. ^7^ ^-^ ^15^

 Although animal studies have shown some progress in pharmacologic therapies, current non- surgical therapies for PTOA of the ankle are mostly palliative and limited to significant adaptation of lifestyle. ^16^ ^-^ ^20^ Progression of early stage PTOA associated with malalignment or instability of the ankle mortise may be successfully halted with corrective osteotomies and ligament reconstruction in carefully selected patients. ^6^ As the disease progresses, salvage options are limited to either joint distraction, arthrolysis, fusion or joint replacement. ^19^ Treatment modalities for focal or confined disease include bone marrow stimulation techniques, cartilage transplantation, osteochondral grafts, collagen membranes, and autologous chondrocyte implantation. The results of these techniques have been reported mainly for osteochondrosis dissecans, not PTOA, and repeated histological analyses have mostly revealed the formation of “hyaline like” fibrocartilage. ^6^ ^,^ ^19^ ^,^ ^20^

Our purpose is to increase medical awareness based on scientific evidence of pathophysiology, molecular biology, and treatment of post-traumatic ankle osteoarthritis.

## METHODS

 The study was approved by the institutional review board of our institutions and did not involve animals nor humans. To support the perspectives of the experts, evidence from the scientific literature respected the Prisma guidelines, and the Picos search strategy was employed as follows: we conducted a search of the relevant scientific literature from January 1, 1977, to September 21, 2023, using databases, including PubMed, MedLine, and Scopus, and gray literature sources. Searched terms were “posttraumatic osteoarthritis” AND “ankle fractures”, “posttraumatic osteoarthritis” AND “cartilage”, “posttraumatic osteoarthritis” AND “synovia”, “posttraumatic osteoarthritis” AND “synovial fluid” NAD “molecular” AND “biology”. Table 1 lists the keywords used in the search. We included case-control, cohort, experimental studies, and case reports, written in English. The selected references were reviewed by all authors and judged on their contribution to the body of knowledge of this topic. A total of 282 abstracts were screened, 114 of which were directly related to the posttraumatic osteoarthritis after malleolar fracture ( Table 2 ). Different aspects of ankle PTOA were extensively detailed below. 

**Table 1.** Keywords used to search the PUBMED database.

**Keywords for PUBMED, MedLine and Scopus literature search**		
Malleolar fracture	Ankle Fracture	Posttraumatic osteoarthritis
Cartilage	Chondral tissue	Synovia
Synovial fluid	Joint damage	Histological changes
**Subheadings used for PUBMED, MedLine and Scopus literature search**		
Cytokine	Matrix metalloproteinases	Physiopathology
Inflammatory cells	Etiology	Collagen
Metabolism	Diagnosis	Chondrocyte
Viability	Treatment	Outcomes

**Table 2.** Types of studies and casuistic resulting of database search.

Animal studies (in vivo/ in vitro)	32
Cadaveric studies	4
Case series	37 (total cases: 1158)
Case control studies	8 (total test cases: 418)
Cohort studies	3 (total patients: 4827)
Review of literature	31
Total selected studies	114
Total patients	6.403

### Understanding the pathophysiology and associated injuries of ankle PTOA

 Hintermann et al ^7^ . Evaluated the arthroscopic findings in acute fractures of the ankle in 288 consecutive patients. Cartilage lesions were found in 228 ankles (79.2%), more often on the talus (69.4%) than on the tibial plafond (45.8%), the fibula (45.1%), and the medial malleolus (41.3%). The frequency and severity of the lesions increased from Weber type-B to type-C fracture. A possible explanation for the disparity between prevalence of chondral defects and the development of ankle arthritis might be that ankle joint cartilage has the higher glycosaminoglycan (GAG) content compared with cartilage on other locations, which yields a high stiffness to the borders of chondral lesions. Furthermore, ankle joint cartilage is the metabolically most active cartilage in the human body, having the highest turnover of aggrecan proteins. 

Although several in vitro studies have demonstrated that an organ system response to acute intraarticular fracture, no established gold-standard is available to identify patients at high risk of PTOA development following an intraarticular fracture. A recent in vivo study showed that synovitis scores and CD68+ macrophages abundance were significantly greater in tissue from patients with ankle fracture compared with normal ankles. More importantly, pro-inflammatory cytokines TNFα, IFNγ, IL-6, IL-8, IL-10, and IL-1β were increased in synovia from patients with ankle fracture in comparison with knee OA, showing that acute inflammation could be more pronounced soon after an ankle fracture than an already installed OA. Notably, IL-6 concentration was significantly increased in the serum of patients with ankle fracture compared with normal serum. Its level also correlated positively with the IL-6 intraarticular concentration, suggesting that it may be a candidate for monitoring early local and systemic inflammatory changes following ankle fractures.

The damage of cracks to the adjacent chondrocytes have been shown in a canine model through the expression of pro-inflammatory markers such as TNFα and IL1β, loss of proteoglycan, and cell cloning immediately after impaction and up to 1 year, at which time some repair has been observed. In a human study, traumatic fracture of an articular surface resulted in significant chondrocyte death and apoptosis, with cells along the edges of the disrupted articular cartilage showing decreased viability and increased apoptosis when compared to those located away from this site. Importantly, a privileged area of viable cells is generally present in the middle and deep zones away from the fracture edge, which suggests that attempts to anatomically reconstruct traumatized joints are reasonable, as vital tissue is present in those areas and might heal the fracture or osteotomy site.

### Chondrocyte death – necrosis or apoptosis?

Chondrocyte dysfunction and death are critical findings of articular degeneration. However, there is questioning on whether there is necrosis or apoptosis of the cell. Increased incidence of chondrocyte death has been found in various forms of joint arthrosis and has been shown to occur in response to mechanical injury. Necrosis is marked by increased uptake of fluids by the cell, with decrease of proteoglycan biosynthesis and increase in water content. The resultant swelling causes rupture of the cell membrane and release of the cell contents. Necrosis of impacted chondrocytes has been linked to overproduction of reactive oxidant species (ROS). It was hypothesized that the chondrocyte mitochondria were the most likely source of the damaging acute impact- related ROS.

Apoptosis results in chromatin condensation, DNA fragmentation, cell shrinkage, and plasma membrane blebbing. In most cases, once the cell membrane integrity is lost, these nuclear and cytoplasmic fragments are taken up by phagocytic cells in the area. The final pathway for apoptosis involves a complex proteolytic system identified as caspases. These proteases activate pro-inflammatory cytokines and ultimately depend on activation of Caspase-3 to complete the autophagic process.

Apoptosis rates in normal cartilage are less than 1% and in osteoarthritis these rates have been reported to average 15%. In the first study to evaluate chondrocyte death after in vivo impact and fracture in humans, the apoptosis rates were more than twice this value, in fact up to 35%. A classic correlation between chondrocyte apoptosis and PTOA includes the production of nitric oxide (NO) and nitric oxide synthase (NOS). As chondrocytes age, there is an increase in the production of NOS, which catalyzes the formation of NO. NO has been shown to decrease chondrocyte production of proteoglycan and type II collagen, making the cartilage less efficient at damage repair and disrupting normal homeostasis. NO has also been shown to cause damage to DNA strands and to trigger apoptosis.

There are investigations of the relationship between cartilage injury and chondrocyte apoptosis. A dose-response was present between the incidence of apoptosis and loading magnitudes. Some investigators also found little apoptosis in their human specimens in the first 3 hours after injury. However, beginning at 6 hours and continuing until 96 hours after injury, there was a consistent increase in apoptosis. Although there is evidence to support the concept that chondrocytes can be stimulated to undergo apoptosis in response to injury, so far little evidence exists to support the claim that chondrocyte apoptosis contributes significantly to the development of posttraumatic osteoarthrosis.

Subchondral bone thickening, severe erosion of the cartilage at the fracture site and acute synovitis within 7 days were also observed. Some authors have investigated chondrocyte death in intraarticular fractures created with realistic impulses in explanted whole joints from cows, pigs, and humans. They reported that the majority of chondrocyte deaths occurred in proximity to the fracture lines in all three species. Remarkably, human ankle joints exhibited dead chondrocytes along the crack lines.

### The mechanisms involved in the development of ankle PTOA - focus on molecular biology

Proprioception normally provided by afferences from the ankle capsule and ligaments may further impair the ability of dynamic stabilizing structures, such as peroneal muscles, thus leading to functional ankle instability. In the further course, disuse atrophy and residual muscle weakness will add to the kinematic disbalance. Pain and fear of re-injury may also inhibit muscle function and lead to stiffness and capsular fibrosis. Alterations in this microenvironment have been shown to induce changes in chondrocyte gene expression and ultimately propagate the initial injury. Other factors beyond alterations in cartilage loading patterns, like adaptability of chondrocytes to the action of inflammatory cytokines in the joint space, must be taken into account in the development of PTOA.

 Immediately after the initial trauma, articular chondrocytes are influenced by various inflammatory chemokines, including TNF- *a* , IL-1 *b* , and MMP-13. Chondrocyte apoptosis continues to increase in the days following trauma and the percentage of dead chondrocytes demonstrated a progressive increment from 6 hours to 5 days post-injury. The caspase apoptotic phenomenon is linked, among others, to high intraarticular levels of TNF- *a* . In a pioneer study, proteoglycan synthesis was markedly suppressed after intraarticular injections of IL-1 *b* in naive mice, whereas higher doses of TNF- *a* were needed for this. Notably, normalization of PG synthesis was shown by blocking of IL-1 with anti-IL-1, thus minimizing cartilage damage. 

Catabolin/IL-1 preparation caused extensive loss of proteoglycan, and later collagen, from the cartilage explant while proteoglycan appeared in the culture medium. It was concluded that the role of Catabolin/IL-1 in vivo is similar to its effects in vitro on cartilage in organ culture. In an earlier study, the same author also stated that synovium affected the cartilage in two ways: first, by a direct action on the matrix, possibly by secreted proteinases, like collagenase, and second, by an indirect action mediated through the living chondrocytes which produce acidic proteinases of the lysosomal system.

 Besides activation of inflammatory response, mechanical damage during traumatic injury has been shown to change gene expression in chondrocytes, activating various degradative enzymes such as MMPs. These MMPs degrade extracellular matrix (ECM) proteins like collagens and GAGs, which in turn accumulate and cause a positive feedback cycle, with further MMP release and progressive cartilage loss, even after cessation of the mechanical injury. Adams et al. demonstrated that pro-inflammatory cytokines IL-6, IL-8, MMP-1, MMP-2, and MMP-3 were significantly elevated in the synovial fluid of fractured ankles when compared to matched contralateral uninjured side, even 6 months after the initial fracture ^14^ . Godoy et al. recently analyzed cellularity and synovial profile in patients with acute ankle fractures in comparison with fresh cadaveric ankles. Cytokine concentrations in synovial fluid samples were significantly higher in the fracture group, especially for IL-2, IL-6, IL-10, and IL-17. Synovial tissue of study group showed an accumulation of collagen and proteoglycan. Another auhor revealed significant elevation of 19 amino acids metabolites in fractures ankles in comparison to the contralateral sides. Among these, glutathione metabolism exhibited the most elevated increase, indicating a possible role for oxidative stress in ankle fractures. A decrease in synovial fluid SF lubricin was also associated with elevation of inflammatory cytokines in human knees after ligament injuries, which may play the joint at a risk of wear-induced damage. 

PTOA can be considered a “whole joint” disease in which synovial reaction can lead to synthesis and release of a wide variety of cytokines and chemokines. Some of these inflammatory mediators are detected in joint tissues and SF in OA and have catabolic effects on chondrocytes. The synovial membrane is also a source of pro-inflammatory and catabolic products, including metalloproteinases and aggrecanases, which contribute to articular matrix degradation.

The influence of the synovium on chondrocytes is triggered by the release of cytokines and growth factors such as IL-1, IL-6, and TNF, stimulated primarily by direct contact of SF and bone marrow. These factors are produced in the synovial membrane and diffuse into the cartilage via the SF and, among other mechanisms, lead to increased apoptosis of chondrocytes. Consequently, synovitis is associated with greater symptoms such as pain and degree of joint dysfunction. Two potential innate inflammatory mechanisms that may lead to synovitis in OA have been suggested: toll-like receptors (TLR) pathways and the complement cascade.

Matrix fragments and products released during cellular stress like PGs and GAGs can activate the innate immune response via pattern-recognition receptors known as TLR. The following cellular response culminates in activation of specific transcription factors, with nuclear-factor κB (NF-κB) playing a prominent role. Many MMP, particularly MMP-13, implicated in OA-related cartilage damage are dependent on the activity of NF-κ B as well.

TLR activation in the synovium is an important stimulus for NFκB activation and subsequent production of chemokines (e.g. IL-8 and CCL5) and cytokines (e.g. IL-1, IL-6, and TNF), which recruit and activate macrophages, granulocytes and lymphocytes. In cartilage, TLR-2 and TLR-4 are up-regulated specifically in damaged areas in patients with PTOA. A more specific study demonstrated that TLR2 and TLR4 signals are important in mediating catabolic responses and in increasing MMP-3 and MMP-13 production, also stimulated by the synovium.

The second pathway to synovitis damage involves complement activation, which is physiologically essential for effective clearance of many pathogens and damaged host components. When complement activity is dysregulated it can lead to extensive tissue damage. Clinically, increased synovial complement component deposition (C3a and C5b-9) in the setting of acute flare-ups of symptomatic OA has been demonstrated, suggesting that complement activation occurs early in the joint during PTOA development.

Activation of both TLR and the complement cascade in the synovium results in transcriptional activation of genes involved in the development of inflammation, most notably genes for soluble mediators such as cytokines and chemokines. These mediators may be produced by a variety of cell types, including macrophages, chondrocytes and synovial fibroblasts. IL-1, IL-7, and TNF are some of the dominant activators of chondrocyte-mediated catabolic protease production, leading to production of MMPs and PG loss via chondrocyte specific receptors. Consequently, these inflammatory mediators represent a potential target for therapeutic interventions against structural joint damage.

Up-regulation of genes encoding acute pro-inflammatory markers has been observed both after acute traumatic injury and PTOA development. Enriched pathways associated with the up-regulated genes include heme biosynthesis and complement cascade. Sebastian et al. investigated the genetic basis of enhanced OA susceptibility in three mouse strains with varying susceptibility to OA: STR/ort (highly susceptible), C57BL/6J (moderately susceptible), and MRL/MpJ (not susceptible). They identified 944, 2330, and 2702 genes differentially regulated in MRL/MpJ, C57BL/6J, and STR/ort, respectively, in response to ACL injury, including B4galnt2 (beta-1,4-N-acetyl-galactosaminyl transferase 2) and Tpsab1 (tryptase alpha/beta 1). Up-regulation of genes encoding both acute pro-inflammatory markers, such as inducible nitric oxidesynthase (iNOS), IL-6, and IL-17, and matrix degrading enzymes, such as ADAMTS-4 (A-disintegrin and metalloproteinase with thrombospondin motif 4) and MMP3, was detected in femoral cartilage, concomitant with extensive cartilage damage and bone remodeling over 21-days post-injury. In injured knees, iNOS, ADAMTS-4, MMP-3, and IL-6 were elevated 19.5-fold, 7.7-fold, 10.2-fold, and 36-fold, respectively at 4 hours post-ACL rupture.

In vitro cartilage injury models have provided much of our basic understanding of the acute effects of impact load on cartilage. However, these models are limited, as they neither represent a natural physiologic environment nor investigate cartilage response to injury over time.

### Detection of early PTOA

**Figure 1.**
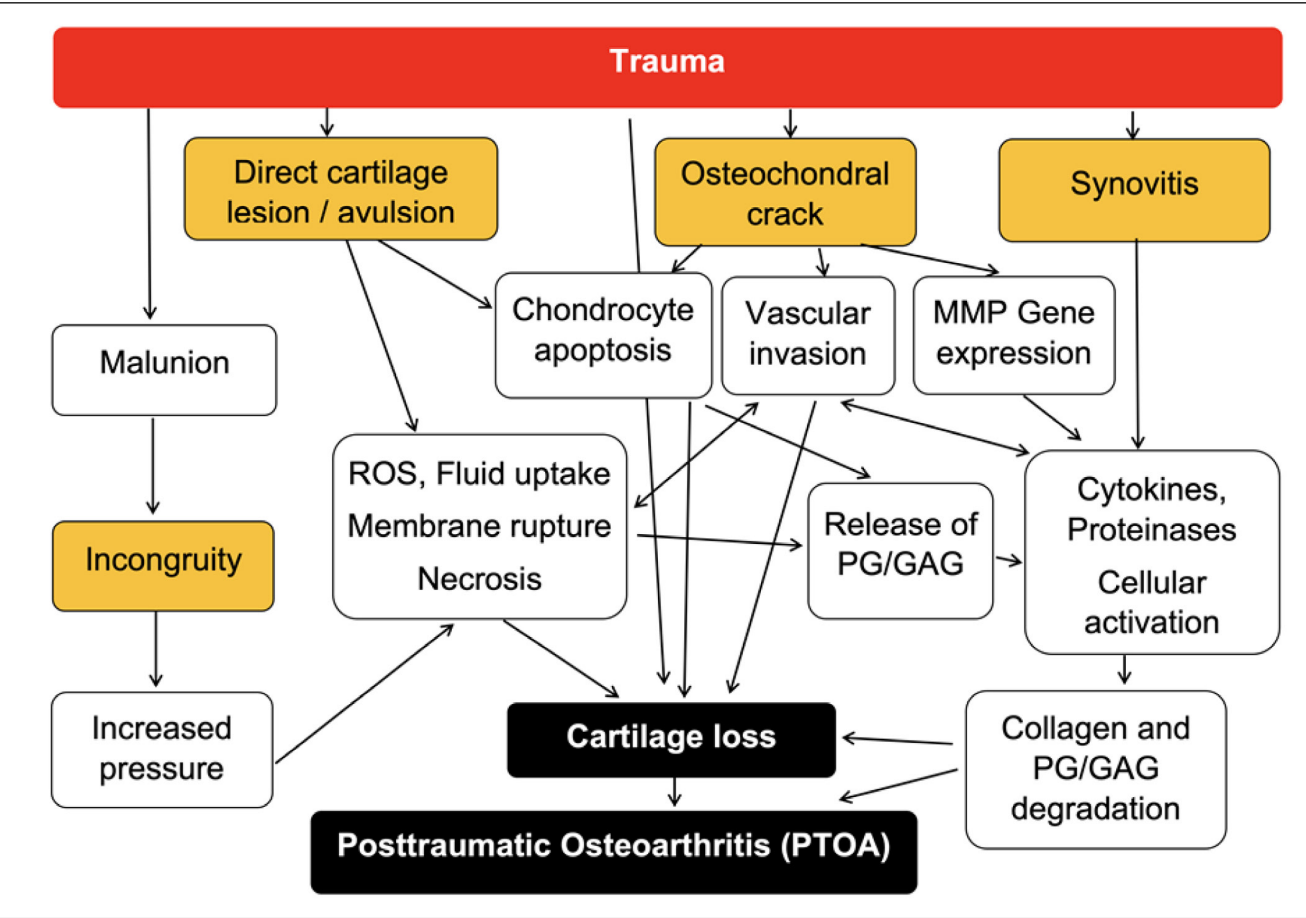
*The holistic approach for understanding different individual evolution and guiding clinicians to an individualized treatment target* .

 Due to the complexity of mechanical, inflammatory, and molecular factors involved in the ankle PTOA and their interactions, it is difficult to allocate the patient into an isolated phenotype. Rather than treating PTOA of the ankle as a single disease, a more holistic approach may overcome different individual evolution and guide clinicians to better target their patient. ( Figure 1 ) 

 The most appropriate modality for early detection of OA in younger patients is magnetic resonance imaging (MRI). Newer techniques like cartilage mapping are capable to detect early alterations in cartilage microstructure, composition of extracellular matrix and biomechanical of chondrocytes. T1ρ is an important modality for evaluation of proteoglycan content, while collagen organization is better visualized in T2 relaxation times. ^6^ T2 mapping has reduced sensitivity to evaluate deep layers of cartilage, since its highly organized structural properties results in extremely short T2 relaxation times. In this context, ultrashort echo time (UTE)–T2 is more sensitive to accurately visualize collagen integrity and cartilage degeneration. 

Single-photon emission computed tomography/computed tomography (SPECT-CT) to evaluate the extent of degenerative changes and their biological activities has been used in patients with PTOA of the ankle. This imaging modality is a combination of bone scanning and CT imaging data and has demonstrated significantly higher interobserver and intraobserver reliability than measurement using CT alone or CT and bone scanning together. Also, SPECT-CT imaging allows to accurately verify the effects of mechanical malalignment on cartilage. Malaligned ankles into varus showed significantly higher radioisotope uptake in the medial joint compartment than in the lateral compartment, whereas malaligned ankles into valgus showed significantly greater uptake in the lateral areas.

 Since OA is an inflammatory disease process, biomarkers of inflammation may be the earliest signs of PTOA. They can be measured in blood, urine, and synovial fluid. TNF- *a* , IL-1, and MMP have all been targeted but the best predictor is yet to be established. However, the same markers are expressed in the wake of an acute fracture as measured with gene expression analysis, and more recently proteomics and metabolomics from microdialysis sampling may also be quantified. Collagen II precursors and metabolites are more specific markers of chondrocyte metabolism. In spite of all that, there is a need for reliable biomarkers to provide prognostic information and to monitor clinical response. Prospective studies correlating patient outcomes with changes in biomarkers profile would be beneficial to guide OA treatments. 

### Perspectives on systemic treatment

Unfortunately, options to treat PTOA are mostly limited to late procedures such as bone marrow stimulation, bulk osteochondral grafting, arthrodesis or joint replacement. Ways of modulating the physiologic response to trauma and potentially preventing chondrocyte death are further described.

In early stages of asymmetric arthritis resulting from malunions, joint-preserving osteotomies can avoid the need for arthrodesis or arthroplasty. Dislocations should be reduced promptly because they increase the soft tissue damage and there is a close correlation between the length of joint dislocation with apoptosis of chondrocytes. In an experimental OA model, intermittent treatment with teriparatide (parathyroid hormone) was found to improve the microstructure, remodel the subchondral bone, and prevent progression of cartilage damage, which may prevent joint collapse by suppressing osteoclastic activity in the subchondral bone.

Statins can potentially modulate the function of chondrocytes. Lovastatin significantly promoted proliferation and inhibited the IL-1b induced apoptosis in rabbit chondrocyte. In contrast, NSAIDs inhibit in vitro chondrogenesis from mesenchymal stem cells. Once chondrocytes are exposed to NSAIDs, their cell cycle was found arrested in the G(0)/G(1) phase. However, under inflammatory conditions the response is different. Celecoxib and indomethacin significantly reduced the number of trauma-induced apoptotic chondrocytes in a study with human articular impacted cartilage. Hyaluronan (HA) protects against chondrocyte apoptosis during the development of OA. Low molecular weight hyaluronan, binds to its specific anti-apoptosis receptors and exerts high expression levels of COL2A1 and AGG genes in OA cartilage.

First evidence exists that bisphosphonates can inhibit chondrocyte apoptosis and degradation of cartilage secondary to corticosteroid-induced apoptosis. Growth factors are important in the physiology of chondrocytes and transforming growth factor (TGF-b) and IGF-1 were found to have anti-apoptotic effects. Similarly, bone morphogenetic protein (BMP-)2 prevents apoptosis of chondrocytes via inhibition of caspase-3 and -9 and increase in Bcl-xL expression.

 Anti-inflammatory interleukins such as IL-4, IL-10, and IL-13 are expressed by articular chondrocytes and have chondroprotective effects. IL- 10 stimulates COL-II and PG expression, and inhibits cytokine induced MMP and NO expression. IL-10 further antagonizes the apoptotic pathways of IL-1b and TNF- *a* and inhibits the levels of pro-inflammatory cytokines. In vitro experiments on chondrocytes have shown that caspase-1 selective inhibitor Z-YVAD blocked chondrocyte apoptosis when exposed to collagenase. The intra-articular application of the pan-caspase inhibitor Z-VAD-FMK in vivo significantly reduced cartilage degradation both macroscopically and microscopically. Similarly, when chondrocytes were subjected to high mechanical stresses or after impaction injuries, caspase inhibitors were found to prevent their apoptosis. 

Diacerein, an inhibitor of IL-1b, reduced OA chondrocyte DNA fragmentation and death through a decrease in the level of caspase-3 expression, levels of iNOS, and secondarily to NO production. Diacerein was found to reduce the pain and symptomatology in patients with PTOA and had an impact on the abnormal subchondral bone metabolism by reducing the synthesis of resorptive factors and osteoclast formation. Curcumin inhibited IL-1b-induced apoptosis and caspase-3 activation in chondrocytes. Recent work has shown that curcumin protects human chondrocytes from the catabolic actions of IL-1b, including MMP-3 upregulation, inhibition of collagen type II, and downregulation of beta1-integrin expression. Resveratrol reduced the SNP-induced apoptosis rate of chondrocytes and level of NO in the synovial fluid. The combination of curcumin and resveratrol had a more potent effect as compared to monotherapy with each drug individually.

Lastly, platelet rich plasma (PRP) significantly increased the proliferation of chondrocytes and decreased their apoptosis in an in vitro study. PRP application resulted in a dose-dependent significant decrease in MMP-3, MMP-13, ADAMTS-5, IL-6, and COX-2, while TGF-b, aggrecan, and collagen type 2, TIMPs and intracellular anti-inflammatory interleukins IL-4, IL-10, IL-13 were increased.

## CONCLUSION

The pathophysiological factors involved in posttraumatic ankle osteoarthritis, such as biological, structural, mechanical, and molecular changes must be studied together, as the interaction between these factors determines the risk of PTOA progression.

Inhibition of a single catabolic molecule or cascade probably is not sufficient to alter the natural progression of the pathological process.

Developing methods to prevent the advancement of PTOA may delay or eliminate the necessity for arthrodesis or arthroplasty, which occurs undesirably earlier in patients with ankle PTOA.
